# The Wild Women of Mortimer Hall

**Published:** 1919-11-01

**Authors:** 


### THE EDITOR'S LETTER-BOX.

**[Correspondence on all subjects is invited, but we cannot in any way be responsible for the opinions expressed by our correspondents, who must give their name and address as a guarantee of good faith, but not necessarily for publication. Correspondents are reminded that brevity of style and conciseness of statement greatly facilitate early insertion.]**

### THE WILD WOMEN OF MORTIMER HALL.

### SPEAKERS CONFINED TO THEM.

*To the Editor of* The Hospital.

Sir,—I attended the meeting held at Mortimer Hall on Saturday. There were several points I should like to have brought before the meeting, but I was refused a hearing. As there were many other nurses in the same position as myself, I hope you will allow the following important points to have publicity in your valuable paper.

What is the position of the "Sick" in the event of a strike being called?

The nurses' employer is either the "Sick," as in the case of the private nurse, or the public in the case of the nurse in the institution. Do nurses think that they are sufficiently essential to the public to ensure that the untrained worker will not readily be employed by it?

The "strike" is the lawfully recognised weapon of a trade union; if, however, it is guaranteed not to make use of the "extreme measure," why register as a trade union?

If the trade union was started with the intention of the "No Strike" policy, as was suggested by some speakers at the meeting, who is to guarantee that this policy will hold good in the future? It was definitely advocated as a "threat weapon," which should certainly be used if necessary. Next year "extremists" may predominate amongst the re-elected officers, or the present officers may develop more extreme views. A trade union would stand between the nurse and her patient, and the nurse must be deprived of individual action. She must obey the trade union regulations or be black-legged.

Of the nineteen objects stated as the aims of the trade union, fifteen are included in those of the College of Nursing, and are already in operation. Why multiply existing organisations? Would it not be better to co-operate with those already established? Success would thus be ensured.

It was regrettable that the meeting was so conducted that one side only was given a hearing, and it was felt that many nurses were carried away by emotion and the excitement of the moment, without being given time to think for themselves or to hear arguments for and against such a step. They were not given the chance to realise what a trade union organisation as applied to the nursing profession might lead to. In fact, one soon realised that the meeting was an organised attack on individuals and on existing organisations, rather than an effort to benefit nurses as a body.

I remain, yours faithfully,

Demobilised Nurse.

